# Double seropositive neuromyelitis optica associated with COVID-19: A case report

**DOI:** 10.3389/fneur.2022.1004132

**Published:** 2022-10-19

**Authors:** Dana Antonescu Ghelmez, Adriana Moraru, Florian Antonescu, Altay Sercan Chelmambet, Amanda Ioana Bucur, Sorin Tuţǎ

**Affiliations:** ^1^Department of Neurology, “Carol Davila” University of Medicine and Pharmacy, Bucharest, Romania; ^2^Department of Neurology, National Institute of Neurology and Neurovascular Diseases Bucharest, Bucharest, Romania

**Keywords:** neuromyelitis optica, anti-AQP4 antibodies, anti-MOG antibodies, COVID-19, NMOSD

## Abstract

Neuromyelitis optica spectrum disorders are characterized by severe demyelination and axonal damage with autoimmune mechanisms, predominantly targeting the optic nerves and the spinal cord. Patients often test positive for anti-AQP4 antibodies, while some have anti-MOG antibodies. Double seropositivity has been described, with a variable prevalence (0 to 26%) dependent on the testing method. The clinical significance of double seropositivity remains unclear. We present the case of a 65-year-old patient, admitted to our clinic with optical neuritis, followed up approximately 10 days later by cervical myelitis, who tested positive for both anti-AQP4 and anti-MOG antibodies. The clinical onset coincided with a mild form of SARS-CoV-2 infection. The neurological symptoms were initially relatively subdued, which delayed the diagnosis. The patient was not vaccinated against SARS-CoV-2. The clinical picture was compatible with an anti-AQP4 phenotype. The patient was started on corticosteroid therapy, under which the clinical response was good. Our case reinforces the idea that SARS-CoV-2 can precipitate autoimmune demyelinating diseases since SARS-CoV-2 infection has already been demonstrated as a risk factor for NMOSD relapses. To the best of our knowledge, this is the first reported case of double seropositive neuromyelitis optica associated with COVID-19. We expect that in the near future, as the true burden of COVID becomes clearer, we shall encounter other cases which can trace their apparent clinical onset to a SARS-CoV-2 infection. Careful attention should be paid to the apparent minor neurological symptoms of COVID-19.

## Introduction

Neuromyelitis optica spectrum disorders (NMOSD) are rare autoimmune disorders characterized by severe demyelination and axonal damage, predominantly targeting the optic nerves (ONs) and the spinal cord (1). The AQP4-IgG serum antibodies play a direct role in the pathogenesis of NMOSD, targeting a water channel protein found in high concentrations in the astrocytic foot processes. A small percentage of ~12% of patients who fulfill the clinical criteria for NMOSD are seronegative for AQP4-IgG (2). Approximately 40% of seronegative patients have anti-myelin oligodendrocyte glycoprotein (anti-MOG) antibodies (3). Whether an anti-MOG disease is a distinctive clinical entity is still to be determined, but some common characteristics seem to be found more frequently in this group: a simultaneous manifestation of optic neuritis and myelitis at onset, simultaneous bilateral optic neuritis, myelitis more often affecting the lower portion of the spinal cord, and monophasic attack or fewer relapses than the AQP4-IgG seropositive patients (1, 4). SARS-CoV-2 is a new pathogen that has been shown to have significant interactions with the immune system, not only in the acute setting but also in the long term, exacerbating or initiating numerous autoimmune disorders (5).

## Case presentation

We present the case of a 65-year-old Caucasian male, without any significant medical history, admitted to our clinic with bilateral lower limb motor deficit and distal paresthesia in all limbs, with an onset of ~3 weeks prior. The patient also complained of sudden bilateral decreased visual acuity, more severe on the right side, which preceded the motor deficit by approximately 10 days and had a spontaneously favorable evolution and was partially remitted by the time of admission.

An ophthalmologist in another service evaluated the patient approximately 7 days from the onset of the visual symptoms, where he tested positive for SARS-CoV-2. At that moment, he had had mild respiratory symptoms (slight cough and rhinorrhea) for a~2 days. The interval between the onset of the neurological and COVID-19 symptoms was ~5 days.

The ophthalmological examination noted VOS Presbyopia 0.5 sc (0.7 c), VOD PHM, with a regular fundus examination in both eyes. At that time, sight in the left eye was already spontaneously improving, and the diagnosis was right retrobulbar optic neuritis. He was not started on any therapy and received a recommendation for a neurological evaluation and a brain MRI scan. The patient chose to isolate himself at home for 14 days, per the local recommendations at the time.

Approximately 3 days into the isolation period, he developed lower limb paresthesia, followed by paraparesis and ataxia, which progressively worsened until he could no longer walk.

Our initial clinical examination showed a slight loss of visual acuity in the right eye (he could read a newspaper), asymmetric paraparesis (3/5 MRC on the right, 4/5 MRC on the left), bilateral Babinski sign, distal paresthesia in all limbs, severe bilateral lower limb myoarthrokinetic and vibratory hypoesthesia, with important secondary ataxia, and a T10 sensory level on the left side; walking was impossible.

**Figure 1:**
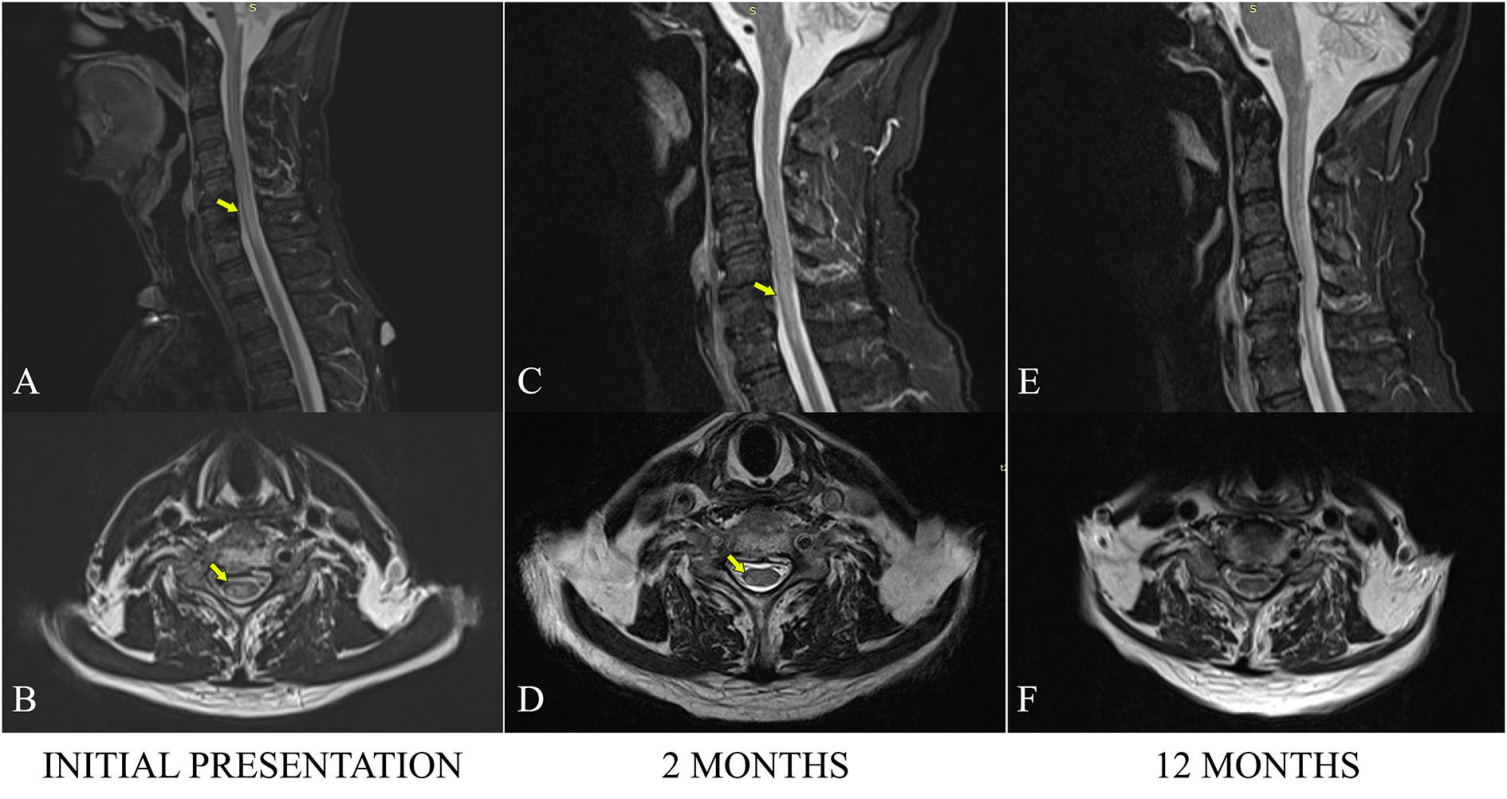
Sagittal MRI STIR sections of the cervical spine at admission **(A)**, 2 months **(C)**, and 12 months **(E)**. Axial MRI TSE sections at approximatively the same level (a plane passing through the upper C6 plateau) at admission **(B)**, 2 months **(D)**, and 12 months **(F)**. The hyperintense STIR cervical lesion is visible, extending from C4 to C7. Progressive fading of the increased signal is visible over time, especially on the axial images.

**Figure 2:**
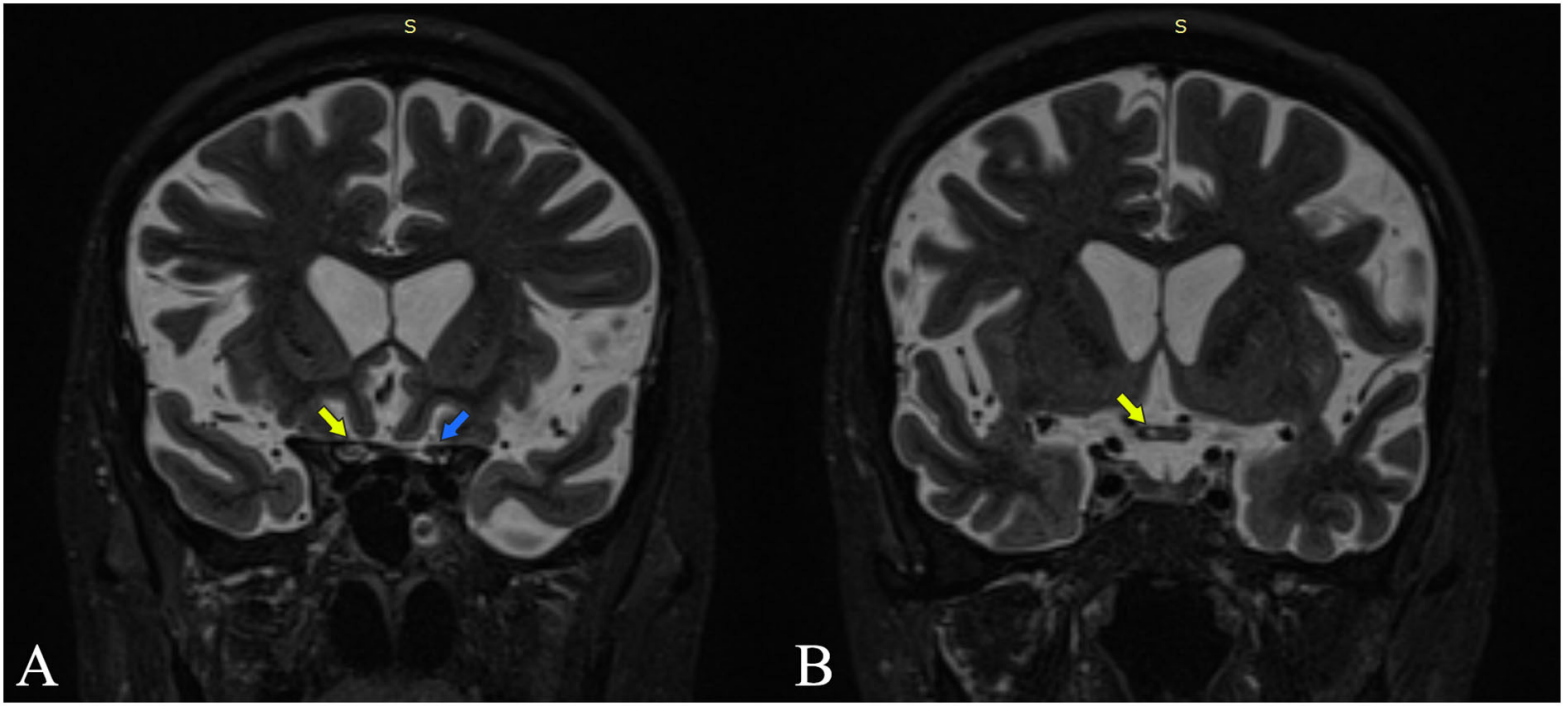
Coronal MRI STIR sections are at the intracranial optic nerves **(A)** and optic chiasm **(B)**. Yellow arrows point out increased STIR signal intensity at the level of the right optic nerve and the right side of the chiasm. The blue arrow points out the left optic nerve with normal signal intensity.

The full spine MRI revealed a non-enhancing, T2 and STIR hyperintense cervical demyelinating lesion extending from C4 to C7 levels (Figures 1A,B), cervical spondylotic changes, and degenerative lumbar stenosis at L4–L5 levels. The cerebral MRI was unremarkable except for a slight STIR hyperintense signal affecting the right ON, extending posteriorly into the optic chiasm (Figure 2).

The patient was evaluated extensively for autoimmune disorders associated with myelitis, testing negative for ANA, c-ANCA, p-ANCA, anti-Ro, anti-La, and anti-neuronal antibodies. The infectious screening was negative except for the incidental presence of IgG against HCV. Testing for HIV, syphilis, and borreliosis was negative. The B12 serum level was normal. Serum anti-AQP4 antibodies and anti-MOG antibodies were both positive. The AQP4 was tested by indirect immunofluorescence with a titer of 1:1,000 (*N* < 1:10). The MOG testing was Western Blot.

The CSF had elevated protein levels (albumin 0.44 g‰, positive Pandy test) without pleocytosis (6 elements/μL) and no oligoclonal bands. The IgG index was normal (0.63).

Given the patient's history of optic neuritis, longitudinal extensive cervical myelitis, positive tests for anti-AQP4 and anti-MOG antibodies, and the exclusion of other possible causes that could have caused central nervous system demyelination, a diagnosis of neuromyelitis optica was reached.

The patient had been treated with iv Methylprednisolone 1,000 mg daily for 5 days with significant improvement of motor function (4-/5 MRC bilaterally) and partial remission of the paresthesia but with the persistence of the objective sensory deficits. The patient was able to walk using a Zimmer frame. He was discharged with oral prednisone at 1 mg/kg/day.

At the 2-month checkup, the patient had continued to improve, presenting with normal visual acuity, asymmetric paraparesis (4-/5 MRC on the right, 4+/5 MRC on the left), bilateral lower limb paresthesia up to the lower half of the thighs, and crural proprioceptive hypoesthesia, still requiring a frame for walking. The cervical MRI showed a slightly favorable evolution of the cervical lesion (Figures 1C,D). As a corticoid-sparing treatment, he was started on Azathioprine (AZA), gradually reaching a dose of 150 mg per day.

He was seen again at the 1-year mark, being stationary from a neurological point of view, with the persistence of the asymmetric paraparesis and sensory anomalies. The cervical MRI examination was discreetly improved, as the intramedullary pathological signal had continued to decrease in area and intensity (Figure 1F). AZA was continued in the same doses while prednisone tapering was initiated.

## Discussion

As the last 2.5 years unfolded, SARS-CoV-2 has repeatedly been shown to initiate and decompensate many autoimmune diseases (6).

This seems to be a family effect for coronaviruses, but SARS-CoV-2 stands out among its peers (7). A recently published review underlines the potential of coronaviruses to spread and persist in the central nervous system (CNS) and their potential for neuropathogenesis. This persistence may be associated not only with the induction or exacerbation of long-term neuropathologies such as multiple sclerosis but may be able to explain persistent neuropsychiatric symptoms associated with long COVID (8).

Although the exact mechanism of virus dissemination in the CNS has not been established, the two possible explanations are either hematogenous spread from the systemic circulation or trans-neuronal spread via the olfactory pathway. In addition, the CNS can be potentially compromised through an ischemic–hypoxic insult of the blood-brain barrier resulting from severe respiratory insufficiency or by immune-mediated mechanisms (9, 10). Anti-AQP4 antibodies have been shown to induce interleukin-6 (IL-6) production in astrocytes, and IL-6 signaling to endothelial cells induces blood-brain barrier dysfunction (11).

The link between SARS-CoV-2 and several neurologic autoimmune pathologies, including Guillain-Barré syndrome, multiple sclerosis, and vasculitis, has also been shown by multiple studies (12, 13).

Although SARS-CoV-2 is known as a risk factor for NMOSD relapses, a causal relationship is more difficult to prove (14). Still, accumulating data support a demyelinating aspect of SARS-CoV-2 infection (9). The ethiopathogenic process is not fully understood. Carlos A et al. hypothesized a possible loss of tolerance to self-antigens caused by a state of transient immunodeficiency of both acquired and innate components (15). According to Wu et al., a neuronal injury may be produced by immune-mediated pathways (16). The binding of the SARS-CoV-2 virus to the ACE2 receptors in the CNS triggers an intense local inflammatory response with impaired blood-brain barrier permeability (10).

Only a handful of cases of neuromyelitis optica with the onset in close temporal relation with a SARS-CoV-2 infection have been reported (17). Most of them tested positive for AQP4 antibodies. To our knowledge, ours is the first case having double seropositivity for AQP4 and MOG.

In NMOSD cohorts, the incidence of double seropositive patients is variable and method-dependent (18–20). In one study, Kezuka et al. found 26% of patients (6 out of 23) to be seropositive for both AQP4 and MOG antibodies (21). However, a study by Sato et al. on a group of 215 patients found no double seropositive cases (22). The discrepancy between the two results can be attributed to the method of detection: cell-based assay in Sato's study vs. ELISA in Kezuka's study. However, in a later study, Kezuka found two double-positive patients with a cell-based assay using full-length human MOG cDNA-expressing HEK cells (23). As mentioned above, in our case, AQP4 antibodies were tested by indirect immunofluorescence and MOG antibodies by Western Blot.

There are notable clinical differences between NMOSD with positive serology for AQP4 compared with MOG-positive cases. Cases with anti-AQP4 antibodies tend to involve the posterior part of the ON or the chiasma. The spinal cord involvement is usually cervicothoracic, while patients with anti-MOG antibodies tend to have anterior involvement of the ON, often bilateral and longitudinally more extensive, and a lower spinal cord involvement (often including the conus medularis) (24).

Regarding chiasmal involvement, a recent study has shown smaller differences than expected between the two groups, with a frequency of 20% in AQP4-positive patients and 16% in MOG-positive cases. In the MOG subgroup, the ON lesion was more often longitudinally extensive (25).

The differences seem to extend to treatment response, but the details are far from clear. Both situations require prompt immunosuppression, and both respond well to Rituximab. MOG-positive NMOSD seems to be more responsive to corticotherapy, with relapses being more frequent on steroid withdrawal (24).

The clinical picture of our patient, with posterior optical neuritis and cervical myelitis, was compatible with the anti-AQP4 phenotype. We believe the patient had suffered from optic neuritis with chiasmal involvement, with a clinical impact mainly on the right eye. This would explain the initially diminished visual acuity in both eyes and was supported by the MRI, which shows an extension of the inflammation in the optical chiasm (Figure 2).

Notably, the optic neuritis had a spontaneous favorable course, and the spinal lesions, while topographically extensive, were invalidating mainly due to the sensory ataxia. A CSF SARS-CoV-2 was not ordered, as it would not have been informative. The patient was admitted to our clinic 3 weeks after the onset, and previous studies have shown that even in cases where the virus can be detected in the CSF, this happens early in the course of the infection. The levels are low and quickly transient (26, 27).

Interestingly, our patient's onset of clinical neurological symptoms overlapped with COVID-19, the rarer of two situations. In the case series, 68% of transverse myelitis associated with SARS-CoV-2 infections had a latency of 10 days up to 6 weeks, suggesting the complications were mediated by the host's immune response to the infection. In the remaining 32%, the latencies varied from 15 h to 5 days, advocating a direct neurotropic effect of the virus (28).

As the spinal cord involvement ensued about 5 days after the debut of the respiratory symptoms of COVID-19 and progressively worsened during the next weeks, the autoimmune mechanism is a certainty. The situation is not so clear-cut regarding the optical neuritis, which had its onset approximately 5 days before SARS-CoV-2 manifested in the form of respiration. The incubation period for COVID-19 is considered to be 4–5 days in most cases, with a maximum of 14 days following exposure (29). The patient was unaware of any contact with known cases of SARS-CoV-2 infection, so we cannot place the exposure date. There have been rare reports of cases of optic neuritis in patients with concomitant SARS-CoV-2 infection and no known autoimmune pathology, but while an acute neurotropic effect of the virus cannot be excluded, the chiasmatic involvement with bilateral diminished visual acuity and the fact that it was rapidly followed by myelitis, argue strongly in favor of an autoimmune mechanism in the context of NMOSD (30).

According to a recent study, more than a third of hospitalized COVID-19 infected patients developed neurological symptoms at some point, but unfortunately, most are not able to undergo extensive imagistic workup in the acute phase (31, 32). Taking into account that, even in our patient who had significantly impaired walking, the diagnosis was delayed for about 3 weeks and that some cases might have an even milder initial phase, we think that in the near future, as the true burden of COVID-19 becomes more clear, we might encounter other cases that can trace their apparent clinical onset to “minor” neurological symptoms following a SARS-CoV-2 infection. This aspect might become even more interesting as these patients get repeated infections with the same or different variants.

## Conclusion

SARS-CoV-2 is known to significantly interfere with the immune processes of the body. Since NMOSD does not have a very high incidence, it took a while longer to become obvious that COVID-19 could be a triggering factor, but indeed this seems to be the case. The main serological and clinical forms appear to be of the AQP4 type.

Acute vision disturbances or distal sensory symptoms should be taken seriously in such patients, even if they are mild, with early diagnosis and treatment being the main way to reduce disability.

The significance of double seropositivity remains unclear, as we do not have sufficient evidence suggesting that both types of antibodies would be simultaneously pathological. Cell-based assays are the gold standard and should be used whenever possible. While serology can be used to orient some aspects of the treatment, double seropositive cases do not have this advantage. In such cases, we think the clinical orientation toward an AQP4 or MOG phenotype can be used instead.

## Data availability statement

The original contributions presented in the study are included in the article/supplementary material, further inquiries can be directed to the corresponding author/s.

## Ethics statement

Written informed consent was obtained from the participant for the publication of this case report.

## Author contributions

AM and DA: writing. FA, AB, and AC: review and editing. ST: editing and supervision. All authors have read and approved the submitted version of the manuscript.

## Conflict of interest

The authors declare that the research was conducted in the absence of any commercial or financial relationships that could be construed as a potential conflict of interest.

## Publisher's note

All claims expressed in this article are solely those of the authors and do not necessarily represent those of their affiliated organizations, or those of the publisher, the editors and the reviewers. Any product that may be evaluated in this article, or claim that may be made by its manufacturer, is not guaranteed or endorsed by the publisher.
